# Velopharyngeal Insufficiency in Submucous Cleft Palate: Importance of Combined Surgical Cleft Palate Repair and Dedicated Speech Therapy

**DOI:** 10.1097/SCS.0000000000013070

**Published:** 2026-06-17

**Authors:** Tim F.P. Ritzen, Maud J.M. Verbeek, Shauni A.D. Caenen-Drummen, Sander M.J. van Kuijk, Darren I. Booi, René R.W.J. van der Hulst, Rutger M. Schols

**Affiliations:** * Department of Plastic, Reconstructive and Hand Surgery, Cleft Team, Maastricht University Medical Centre, MosaKids Children’s Hospital; † Department of Speech Therapy, Cleft Team, Maastricht University Medical Centre, MosaKids Children’s Hospital; ‡ Department of Clinical Epidemiology and Medical Technology Assessment, Maastricht University Medical Centre, Maastricht, The Netherlands; § Department of Plastic, Reconstructive and Aesthetic Surgery, Cleft Team, University Hospital Brussels (UZ Brussel), KidZ Health Castle Children’s Hospital, Free University Brussels (VUB), Brussels Health Campus, Belgium

**Keywords:** Cleft audit protocol for speech-augmented, speech therapy, submucous cleft palate, surgical cleft palate repair, velopharyngeal insufficiency

## Abstract

**Objective:**

This retrospective study aimed to evaluate the different conservative (ie, speech therapy) and surgical treatment approaches of velopharyngeal insufficiency (VPI) associated speech pathology in patients with submucous cleft palate (SMCP), performed within a single tertiary cleft care centre.

**Patients, Participants:**

Thirty-three patients treated for SMCP between January 1999 and December 2023, registered in a database of the Maastricht University Medical Centre and MosaKids Children’s Hospital and a database of the Dutch Association for Cleft Palate and Craniofacial Anomalies (NVSCA), were included.

**Main Outcome Measures:**

The decrease in VPI-associated speech pathology after treatment was retrospectively scored using the Cleft Audit Protocol for Speech-Augmented (CAPS-A) in the Dutch language, based on the Dutch Cleft Speech Evaluation Test (DCSET).

**Results:**

A significant reduction of the sum scores of the CAPS-A Dutch was seen in surgery combined with speech therapy (95% CI: 1.46–4.19) and speech therapy after surgery (95% CI: 0.85–3.48). Speech therapy only lowered the sum scores, but did not show a significant effect (95% CI: −1.30 to 1.30). Both surgical and conservative treatment resulted in a reduction of VPI-associated speech pathology, but only surgery significantly reduced hypernasality (*P*=0.005) and improved speech intelligibility (*P*=0.005).

**Conclusion:**

This study confirms the need for a multimodal treatment approach for VPI in SMCP by combining surgical cleft palate repair with pre- and post-operative specialised cleft speech therapy for optimal speech outcomes.

Submucous cleft palate (SMCP) is a specific type of cleft palate with a frequently delayed diagnosis. It has an overall incidence of 0.02% to 0.08% and constitutes 4.3% to 7.3% of all cleft patients.^1^,^2^,^3^,^4^ SMCP develops as a result of an incomplete union of the mesoderm across the soft palate, which should differentiate into muscle, while the fusion of the ectoderm results in an intact oral and nasal mucosa.^5^

SMCP can best be clinically diagnosed according to the triad of Calnan [bifid uvula, bony notch in the hard palate and separation of the soft palate musculature in the midline (zona pellucida)].^6^

Previous research has found children with SMCP present routinely late (34.3–44.0 mo of age) postponing needed surgery to a mean of 49.7 to 53.4 months^2^,^7^ with a range of 6 months to 17 years.^8^ This could be a result of the fact that children start to speak complete sentences around 24 months of age, making it easier to diagnose velopharyngeal insufficiency (VPI) associated speech pathology.^2^

Depending on the age, a child with SMCP can experience symptoms of feeding difficulties and/or nasal regurgitation, ear problems such as acute otitis media and/or otitis media with effusion, and hearing problems associated with repeated episodes of otitis media.^9^

The most frequent problem of SMCP is velopharyngeal insufficiency (VPI) and its associated speech pathology, occurring in 5 to 88% of the children.^1^,^9^,^10^,^11^,^12^,^13^ In VPI, inadequate velopharyngeal closure during speech, due to defective palatal muscles, causes increased nasal resonance (hypernasality), nasal air emission or nasal turbulence/rustle during speech.^14^,^15^

One of the main goals of surgical treatment of SMCP is to treat VPI, facilitating normal speech development.^16^ To achieve this, early detection, preferably before the age of 4 years old,^17^ is crucial as it provides children more time to develop new physiological speech mechanisms after surgery.^2^,^18^,^19^ As not all children diagnosed with an SMCP experience speech impairments, they should not all routinely undergo surgical cleft repair.^20^ This leads to a discussion of whether it is necessary to operate early or to wait until speech problems arise. Surgery can be performed using various techniques, but the surgical technique with optimal speech outcomes is still being researched. The aim of this retrospective study was to evaluate the different conservative (ie, speech therapy) and surgical treatment approaches of velopharyngeal insufficiency (VPI) associated speech pathology in patients with submucous cleft palate (SMCP), performed within a single tertiary cleft care center.

## METHODS

Approval for this study was obtained from the Institutional Review Board at the Maastricht University Medical Center–Maastricht, the Netherlands (METC 2024-0524).

### Patients

In this study, patients treated for SMCP between January 1999 and December 2023, registered in a database of the Maastricht University Medical Center and MosaKids Children’s Hospital and a database of the Dutch Association for Cleft Palate and Craniofacial Anomalies (NVSCA), were included.

Inclusion criteria were SMCP diagnosis and completed treatment by speech therapy and/or surgical cleft repair. Information on birthweight, prematurity, ethnicity, comorbidities, syndromes, DNA-anomalies, family history of clefts, relatives with clefts, consanguinity, adoption, presence of signs of triad of Calnan, velopharyngeal insufficiency (VPI), otitis media, primary and secondary surgical techniques, postoperative complications, and age at diagnosis and treatment was extracted from patients' medical files.

### Outcome Measures

The primary outcome measure was the decrease in VPI-associated speech pathology (eg, hypernasality, nasal emission) after treatment, evaluated using the Cleft Audit Protocol for Speech-Augmented (CAPS-A) in the Dutch language (based on the original CAPS-A developed by John et al^15^).

### Examination

Physical oral examination and diagnosis of SMCP was performed by the plastic surgeon from the cleft team after referral by an otorhinolaryngologist, general practitioner, pediatrician, speech therapist, or other medical professional. If reported, the signs of the Triad of Calnan^6^ (bifid uvula, bony notch in the hard palate and separation of the soft palate musculature in the midline (zona pellucida)) were extracted from the medical records.

### Syndromes, Genetic Abnormalities, and Comorbidities

Evident syndromes or congenital anomalies are often diagnosed by the pediatrician or midwife present at the delivery room or at the first evaluation of the child after birth. The Maastricht University Medical Center MosaKids Children’s Hospital routinely offers all parents and children genetic testing and counseling.

### Otological Outcomes

Medical records were screened for a history of otitis media and the placement of ventilation tubes.

### Treatment

Treatment of the patients was categorized as follows: no treatment, speech therapy only, surgery with preoperative and postoperative speech therapy, or surgery with only preoperative speech therapy.

Surgical treatments were categorized by surgical technique: intravelar veloplasty (IVV), cranially-based pharyngoplasty, caudally based pharyngoplasty, Furlow palatoplasty, Furlow palatoplasty with buccal fat pad flap, and autologous fat transfer (AFT).

If, during primary surgery, 2 surgical techniques were combined or if there was a need for secondary surgery, this was recorded for each surgery and patient individually. Postoperative complications were classified as fistula (using the Pittsburgh Fistula Classification system^21^) and persistent bifid uvula.

### Speech Therapy

Patients were referred to an extramural speech therapist if indicated by one of the cleft speech therapists at our center. Speech therapy was provided at a frequency of 1 time per week with a gradual reduction towards the end of the treatment, as indicated by the evaluating cleft team speech therapist in consultation with the treating speech therapist. During the treatment period, speech therapy was focussed on treating the phonetic cleft-type speech characteristics and VPI. However, if phonologic speech characteristics were present, these were addressed as well.

### Speech Assessment

In the MUMC+, the speech therapists routinely assess the speech of all children with SMCP according to the Dutch Cleft Speech Evaluation Test (DCSET).^22^ This speech assessment protocol was designed for children with orofacial clefts and is used in all Dutch cleft centers. The DCSET is performed at the minimum age of 2.5 years old, as younger children cannot pronounce sentences, and the test can therefore not be performed.

All speech therapists had specialized training and are certified by the speech pathology working group of the Dutch Association for Cleft Palate and Craniofacial Anomalies (NVSCA) to use this assessment tool. The speech therapists of the MUMC+ are obliged to, and therefore do, participate in the biannual national calibration sessions for the DCSET in order to stay certified.

Preoperative perceptual speech assessment was performed by trained cleft speech therapists working at our center. VPI was diagnosed after a period of 6 months of specialized speech therapy with insufficient treatment results, provided that (on intraoral inspection) a sufficiently long and mobile soft palate is present and the child has been able to be adequately instructed. When perceptual speech assessment revealed signs suggestive of velopharyngeal insufficiency, such as hypernasality or nasal air emission, additional diagnostic modalities, including nasometry, mirror tests, nasoendoscopy, or videofluoroscopy, were performed to objectively assess velopharyngeal function and confirm the diagnosis.

Postsurgery speech and VPI was examined by a specialized cleft speech therapists, again using acoustic nasometry or mirror tests as additional diagnostics. The written reports of both preoperative and postoperative perceptual speech assessments were extracted from the patients’ medical files and retrospectively scored by 2 trained cleft speech therapists (M.V. and S.C.) individually using the Cleft Audit Protocol for Speech–Augmented (CAPS-A) in Dutch language, as described in Wijnants et al.^23^ Extracted speech outcomes included those for speech intelligibility, hypernasality, hyponasality, nasal emissions and nasal turbulence, as well as 12 different cleft type speech characteristics (CTCs).

Speech intelligibility was rated on a 0 to 4 scale, where 0 indicates speech is intelligible and 4 indicates speech is unintelligible. Hypernasality was evaluated on a 0 to 4 scale, with 0 indicating no hypernasality and 4 representing severe hypernasality during the production of vowels and voiced consonants. Hyponasality was scored on a 0 to 2 scale, where 0 indicates absence and 2 indicates marked hyponasality. Nasal emission and nasal turbulence were each scored on a 0 to 2 scale, with 0 indicating absence and 2 indicating frequent occurrence in more than 10% of oral pressure consonants. The scores of these 5 speech outcomes were collected individually and summed (sumscore) for analysis. For the analysis of the results of the CAPS-A Dutch, CTCs were merged into 4 clusters of anterior oral, posterior oral, nonoral and passive characteristics.

Nasometry was performed using the Nasometer II model 6450 (Kaypentax, Montvale, NJ). During the nasometry tests, patients were asked to read aloud validated nasal, oronasal and denasal sentences in Dutch. In Dutch language a nasality score >2SD or <2SD is considered abnormal.

While patients were repeating several oral sounds and/or sentences aloud after the speech therapist, the presence of nasal emissions was objectified by mirror tests. For each nostril, the presence of nasal emissions and whether these emissions were audible was noted.

### Statistical Analysis

Baseline characteristics were summarized using median and interquartile range (IQR) or as count and percentage. Statistical null-hypothesis testing was performed using the paired-samples and independent-samples *t* test for comparison of continuous treatment outcomes over time, and the difference between surgical and conservative treatment outcomes, respectively. The ordinal treatment outcomes of the individual CAPS-A Dutch speech parameters were analysed using the Wilcoxon signed-rank test. To determine inter-rater reliability (IRR), the first postoperative speech assessment was scored separately by each of the speech therapists using the CAPS-A Dutch. In patients who did not receive surgery, the first DCSET was scored. The IRR between the independent assessments of 2 speech therapists was assessed using the Cohen Kappa (κ) for the separate perceptual speech assessment scores and using the intraclass correlation coefficient (ICC) for the sum scores of the same perceptual speech assessment.

All analyses were performed using R Statistical Software (v4.1.3; R Core Team 2022). For all null-hypothesis testing, we used an α of 5%.

## RESULTS

### Patients

Thirty-three patients with SMCP were registered between January 1999 and December 2023 in the databases of the Maastricht University Medical Center and MosaKids Children’s Hospital, and the Dutch Association for Cleft Palate and Craniofacial Anomalies (NVSCA). According to these databases, the incidence of SMCP in the Maastricht University Medical Center constitutes to 7.3% (33/449) of all patients with a cleft diagnosed in this period.

There was a median age at diagnosis and initial treatment by speech therapy of 10.7 years (IQR: 7.1–17.4). The median age at surgery was 4.0 years (IQR: 2.6–6.1). Patients receiving speech therapy only were diagnosed at a later median age, therefore explaining this discrepancy in age between diagnosis and surgery. In total, 4 patients (M:F/2:2) were above 18 years old when presented at our hospital.

Seven (21%) patients (M:F/2:5) were diagnosed with a syndrome. In total 29 (88%) patients were known with a history of (acute) otitis media (with effusion) (M:F/14:15) of which 27 (82%) patients were treated by insertion of ventilation tubes (M:F/14:13).

All patient characteristics are displayed in Supplemental Table 1, Supplemental Digital Content 1, http://links.lww.com/SCS/J736, and 2, Supplemental Digital Content 1, http://links.lww.com/SCS/J736.

### Speech Therapy and Surgical Treatment

In only 5 (15%) patients (M:F/3:2), speech therapy only was sufficient for the treatment of VPI-related speech characteristics. Twenty-six (79%) patients (M:F/10:16) received surgical treatment all preceded by speech therapy. In 4 of those 26 patients, surgery without postoperative speech therapy was sufficient for the treatment of VPI.

Primary surgical treatments performed were: intravelar veloplasty (IVVP) (n=3), cranially-based pharyngoplasty only (n=2), caudally based pharyngoplasty (n=1), Furlow palatoplasty only (n=17), Furlow palatoplasty with buccal pad flaps (n=1), autologous fat transfer (AFT) of the posterior pharyngeal wall and the nasal mucosa of the soft palate (n=1), and cranially-based pharyngoplasty combined with Furlow palatoplasty (n=1).

In 3 cases, revision surgery was needed for which in 2 patients was opted for cranially-based pharyngoplasty. In one patient, a Furlow palatoplasty and a cranially-based pharyngoplasty were combined as secondary treatment. The intravelar veloplasty was performed in another hospital in the Netherlands, but postsurgery follow-up and speech therapy were performed in the MUMC+. An overview of the surgical treatments performed can be found in Supplemental Table 3, Supplemental Digital Content 1, http://links.lww.com/SCS/J736.

Two male patients did not receive any additional treatment, but had had speech therapy in the past. One patient was happy with his previous speech improvement and did not want any additional treatment. The other patient, according to the team’s judgment, would not have benefited from additional speech therapy, was sufficiently satisfied with his speech and did not want any other treatment.

### Speech Assessment

The IRR assessment for the perceptual assessment of M.V. and S.C. showed a Cohen Kappa of 0.863 (95% CI: 0.834–0.891) for the separate and ICC of 0.99 (95% CI: 0.979–0.995) for the sum scores of the CAPS-A Dutch.

An overview of the cleft type speech characteristics, CAPS-A Dutch speech parameters, and mirror test and nasometry results is provided in Supplemental Tables 4–6, Supplemental Digital Content 1, http://links.lww.com/SCS/J736.

Of the 26 patients that received surgery, preoperative and postoperative perceptual speech assessment results were available for 17 patients and only postoperative results for 9 patients. This missing data may reflect the inability of the patient to complete the test, the speech therapist’s judgment that the test was not required based on perceptual speech outcomes or that one of the tests would suffice. One of the 17 patients whose postoperative perceptual speech assessment results were available did not speak enough for the speech therapists to evaluate the different parts of the test sufficiently.

Nasometry results were available preoperatively and postoperatively for 6 patients and only postoperatively for 4 patients. Mirror test results preoperatively and postoperatively, only postoperatively or only preoperatively were available for 2, 7, and 2 patients, respectively.

The median time between the first and last scored evaluations was 1.6 years for both the surgery combined with speech therapy and the speech therapy only group. The median time between evaluations for the analysis of the postoperative effect of speech therapy was 1.4 years.

In the speech therapy only group, there was no significant change in speech parameters after treatment. In the surgery combined with speech therapy group, significant post-treatment improvements were observed in both speech intelligibility and hypernasality. In the overall cohort, both outcomes reached statistical significance (*P*=0.005 for both), while in nonsyndromic patients, the corresponding *P*-values were 0.012 and 0.007, respectively.

In the overall cohort, the evaluation of the effect of surgery and speech therapy on speech outcomes showed a mean decrease in CAPS-A Dutch score of −2.8 points (95% CI: −4.19 to −1.46), *P*<0.001; −0.0 points (95% CI: −1.30 to 1.30), *P*=1.000; and −2.2 points (95% CI: −3.48 to −0.85), *P*=0.003; for respectively the effect of surgery only and surgery combined with speech therapy, speech therapy only, and speech therapy after surgery (Figs. 1-3). The reduction in CAPS-A Dutch sum scores was significantly greater following surgery combined with speech therapy than following speech therapy only (*P*=0.002). As Figure 4 shows, both surgery combined with speech therapy and speech therapy only lowered the CAPS-A Dutch sum scores.

**FIGURE 1:**
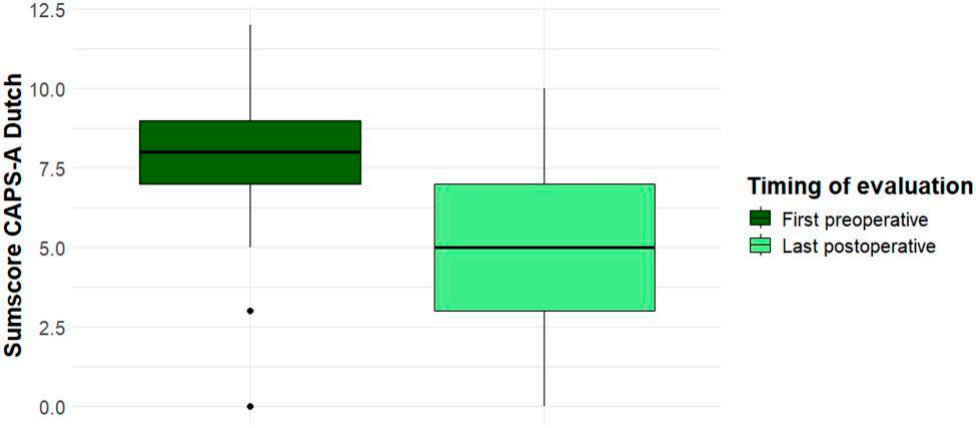
Decrease of CAPS-A Dutch sum score between presurgical and postsurgical speech evaluation. Boxplot showing the CAPS-A sumscores in children with submucous cleft palate, stratified by evaluation moment (the first evaluation before and the last evaluation after cleft palate surgery combined with perioperative speech therapy). Boxes represent the interquartile range (IQR: 25th–75th percentile), with the horizontal line indicating the median. Whiskers extend to the minimum and maximum values within 1.5 times the IQR from the lower and upper quartiles. Values beyond this range are plotted as outliers (black dots).

**FIGURE 2:**
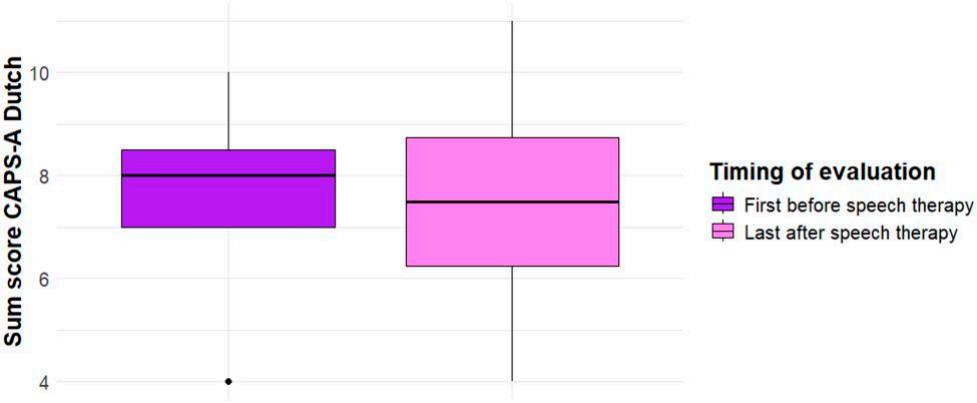
Decrease of CAPS-A Dutch sum score between first and last speech evaluation in the speech therapy only group. Boxplot showing the CAPS-A sumscores in children with submucous cleft palate, stratified by evaluation moment (the first evaluation before and the last evaluation after speech therapy only). Boxes represent the interquartile range (IQR: 25th–75th percentile), with the horizontal line indicating the median. Whiskers extend to the minimum and maximum values within 1.5 times the IQR from the lower and upper quartiles. Values beyond this range are plotted as outliers (black dots).

**FIGURE 3:**
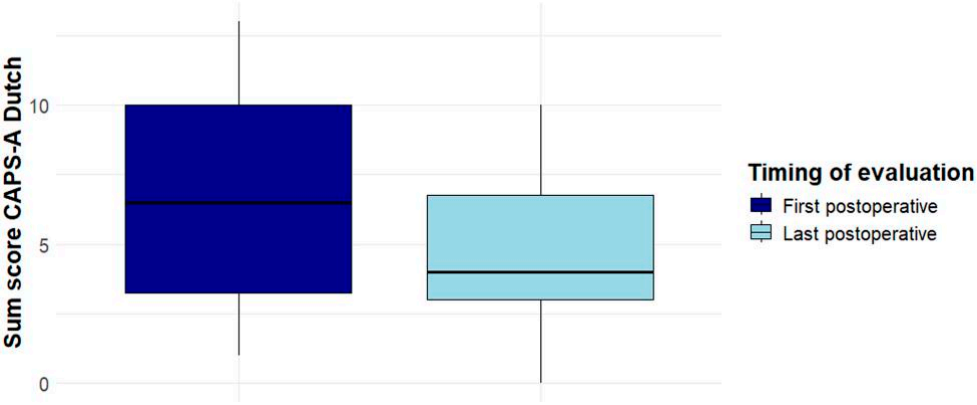
Decrease of CAPS-A Dutch sum score between first and last postsurgical speech evaluation. Boxplot showing the CAPS-A sumscores in children with submucous cleft palate, stratified by evaluation moment (the first and last evaluation after cleft palate surgery and preoperative speech therapy). Boxes represent the interquartile range (IQR: 25th–75th percentile), with the horizontal line indicating the median. Whiskers extend to the minimum and maximum values within 1.5 times the IQR from the lower and upper quartiles. Values beyond this range are plotted as outliers (black dots).

**FIGURE 4:**
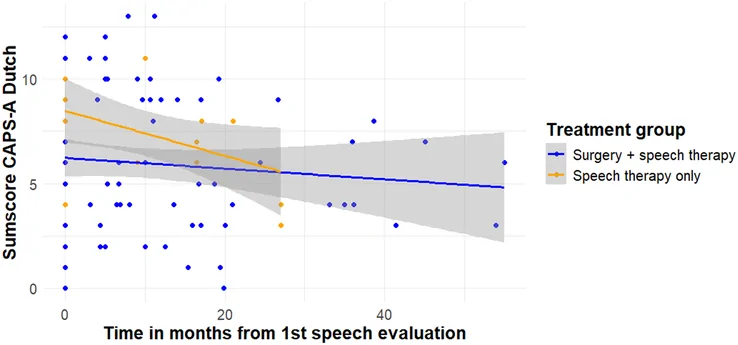
Linear regression of the decrease in CAPS-A Dutch sum score of all patients, divided by treatment. Scatterplot depicting the relationship between time (mo) since a patients first speech evaluation and the CAPS-A sumscore in 2 groups: patients with a submucous cleft palate treated by surgical cleft palate repair combined with speech therapy (blue dots) and patients with a submucous cleft palate treated by speech therapy only (orange dots). Each point represents a different speech evaluation in all patients within the specified group. Linear regression lines are displayed for both groups: a solid blue line for the surgery combined with speech therapy group and a solid orange line for the speech therapy only group. Shaded areas around each regression line indicate the 95% CIs.

Dividing the cohort into syndromic and nonsyndromic patients, nonsyndromic patients only showed a mean decrease in CAPS-A Dutch score of −3.4 points (95% CI: −5.16 to −1.61), *P*<0.001; −0.0 points (95% CI: −1.30 to 1.30), *P*=1.000; and −2.9 points (95% CI: −4.47 to −1.37), *P*=0.001; for respectively the effect of surgery only and surgery combined with speech therapy, speech therapy only, and speech therapy after surgery.

Similarly, syndromic patients only showed a mean decrease in CAPS-A Dutch score of −1.0 points (95% CI: −3.39 to 1.39), *P*=0.252 and −0.2 points (95% CI: −2.42 to 2.02), *P*=0.815, for respectively the effect of surgery only and surgery combined with speech therapy, and speech therapy after surgery. None of the syndromic patients received speech therapy only as treatment.

## DISCUSSION

This single-center retrospective cohort study aimed to evaluate the different conservative (ie, speech therapy) and surgical treatment approaches of velopharyngeal insufficiency (VPI) associated speech pathology in patients with submucous cleft palate (SMCP).

The incidence of SMCP was reported at 7.3%, which corresponds with the Dutch study of Smarius et al.^2^ There was a median age at surgery of 4.0 years (IQR: 2.6–6.1), which is slightly earlier compared with 4.4 to 5.5 years in previous studies.^2^,^24^,^25^,^26^ This could be attributable to this study’s 25-year review period, in which diagnostic and treatment protocols have improved.

Seventy-eight percent of the patients received surgery after a trial of speech therapy. This is in line with 37.5% to 85.7% in recent.^2^,^7^,^18^,^24^,^27^ Furlow palatoplasty, performed in 58% of all cases, was the preferred primary technique, showed good speech outcomes and minor complications. Other articles,^2^,^18^,^26^ show great interest in the use of the IVVP although a recent review suggests the pharyngeal flap to have the best outcomes in first line treatment.^8^ However, this preference for Furlow palatoplasty as the primary technique is confirmed in other publications,^28^,^29^ showing comparable and even higher application of Furlow palatoplasty. Furlow palatoplasty could therefore be suggested as primary surgical treatment, while the cranially-based pharyngoplasty showed good results as a revision technique.

Syndromes were reported in a total of 7 patients of which 3 cases were VCF syndrome which is consistent with other publications.^2^,^7^,^24^,^26^,^30^ In this cohort, syndromes appeared to have a negative influence on the final speech outcomes as the sum and individual speech characteristic scores in the surgery group improved after excluding the syndromic patients. This study supports the idea that cranially-based pharyngoplasty is superior for the treatment of VPI in patients with SMCP and VCF syndrome.^9^ One of 3 patients with VCF syndrome was successfully treated with primary cranially-based pharyngoplasty and another as revision after Furlow palatoplasty. The remaining patient was successfully treated with a Furlow palatoplasty. Bezuhly et al^29^ argue that children with VCF syndrome, compared with regular cleft patients, require ∼2 additional years to obtain normal resonance after surgery and should therefore receive prolonged and intensive speech therapy before considering revision surgery. In this study, the median time between the first and last postoperative speech evaluation was 1,4 years and therefore revision surgery could have been performed before the true effect of primary surgery was present. For surgical treatment of patients with VCF syndrome technique, the cranially-based pharyngoplasty should again be considered.

In evaluating speech, the CAPS-A in the Dutch language proved to be a useful tool for systematically evaluating cleft speech characteristics. Upon visual inspection of the data in Figure 4, both treatments, surgery combined with speech therapy and speech therapy only, were effective in decreasing the CAPS-A Dutch sum scores. Speech intelligibility showed higher scores in the surgery group. It was, however, together with hypernasality, the only speech parameter showing statistically significant improvement after combined treatment with surgery and speech therapy. Speech therapy only did not have a statistically significant effect on any speech parameter, which could be a result of the very small sample size (n=7) of this group. The analysis of the postoperative effect of speech therapy showed a significant decrease of speech pathology, confirming the significance of speech therapy in the multidisciplinary treatment of SMCP.^31^

Dentalisation and interdentalisation were the most observed cleft type speech characteristics (CTCs) in both groups, but showed good improvement after treatment. Some patients presented with nonoral and passive CTCs and showed inconsistent treatment effectiveness. Two patients of the speech therapy-only group newly developed a nonoral CTC after treatment. In the surgery group, 2 patients appeared to developed passive CTC’s after treatment. One patient did not show this CTC at the preoperative speech assessment, but the other patients’ speech was not assessed before surgery. A possible cause could be residual symptomatology of pre-existing VPI and insufficient results of postoperative speech therapy. However, it is not clear whether this could be directly associated to either cleft surgery or postoperative speech therapy. Due to differences in the amount of reports, and nasometry and mirror tests available before and after treatment, no definitive conclusions can be drawn on these assessments.

For an unknown reason, there was a 16 to 34% higher incidence of otological disease compared with other related articles.^26^,^32^,^33^ Twenty-seven patients received transtympanic ventilation tubes, which, based on yet unpublished data on the same cohort, effectively decreased hearing loss and the frequency of otitides occurrence. This effect of ventilation tubes is in line with recent literature.^34^ Although otological disease is reported to cause more speech pathology in children with SMCP,^32^ no such relation could be seen in this study. This could be a result of this studies high incidence of ventilation tube insertion reducing otological disease.

### Limitations

This study has some limitations due to its retrospective nature and small sample sizes for statistical analysis. This study did not examine the effect of the time at diagnosis, surgery or signs of Calnan. Moreover, the speech outcomes were not evaluated separately for each of the surgical techniques performed. The speech outcomes were examined live with a perceptual evaluation tool (the DCSET) and, for research purposes, scored with another tool (the CAPS-A in Dutch language) based on medical records. Although this Dutch version of the CAPS-A is currently undergoing validation, the CAPS-A has already been validated in other Germanic languages such as Belgian Dutch,^35^ British English,^15^ and American English.^36^ In contrast to the DCSET, which evaluates speech on the level of words, the CAPS-A evaluates speech in sentences and should therefore be used in children of a minimum of 4 years old. Furthermore, the CAPS-A is a qualitative instrument and the sum scores can thus only depict the statistical effect of treatment, but not its clinical relevance. Finally, there was too much missing data on nasometry and mirror tests, objective measurements of hypernasality and nasal emission, to analyse those results and to support or deny the results of the perceptual assessments.

## CONCLUSION

In the treatment of VPI in SMCP, the main goal is to facilitate normal speech development by restoring velopharyngeal function. This study confirms the importance of a multimodal treatment approach in SMCP by combining surgical cleft palate repair with preoperative and postoperative specialized cleft speech therapy. A more well-founded recommendation on the optimal treatment for SMCP will require additional extensive and preferably multi-center research into the effect of, for example, various surgical techniques, age at treatment, severity of SMCP, involvement of different syndromes, and the effect of hearing on speech development.

## Supplementary Material

## References

[1] Weatherley-White RC Sakura CY Jr Brenner LD. Submucous cleft palate. Its incidence, natural history, and indications for treatment. Plast Reconstr Surg 1972;49:297–304 5060321

[2] Smarius BJA Guillaume CHAL Slegers J. Surgical management in submucous cleft palate patients. Clin Oral Investig 2021;25:3893–3903 10.1007/s00784-020-03719-1 PMC8137618 33521885

[3] García Velasco M Ysunza A Hernandez X. Diagnosis and treatment of submucous cleft palate: a review of 108 cases. Cleft Palate J 1988;25:171–173 3259168

[4] Crikelair GF Striker P Cosman B. The surgical treatment of submucous cleft palate. Plast Reconstr Surg 1970;45:58–65 10.1097/00006534-197001000-00008 4902843

[5] Sperber GH. Craniofacial Embryogenesis: Normal Developmental Mechanisms 2003;1:29–59

[6] Calnan J. Submucous cleft palate. Br J Plast Surg 1953;6:264–282 10.1016/s0007-1226(53)80060-3 13126438

[7] Jung SE Ha S Koh KS. Clinical interventions and speech outcomes for individuals with submucous cleft palate. Arch Plast Surg 2020;47:542–550 10.5999/aps.2020.00612 PMC7700856 33238341

[8] Gardiner S Bjornson L Pawliuk C. What technique results in the lowest rate of velopharyngeal insufficiency in patients with submucous cleft palate? A systematic review and meta-analysis. Plast Surg (Oakv) 2024;32:226–234 10.1177/22925503221110066 PMC11046285 38681241

[9] Dean KM Leeper LK. Management of submucous cleft palate. Curr Opin Otolaryngol Head Neck Surg 2020;28:410–413 10.1097/MOO.0000000000000673 33105231

[10] Kaplan EN. The occult submucous cleft palate. Cleft Palate J 1975;12:356–368 1058746

[11] Kono D Young L Holtmann B. The association of submucous cleft palate and clefting of the primary palate. Cleft Palate J 1981;18:207–209 6941867

[12] Shprintzen RJ Schwartz RH Daniller A. Morphologic significance of bifid uvula. Pediatrics 1985;75:553–561 3975126

[13] Ysunza A Pamplona MC Mendoza M. Surgical treatment of submucous cleft palate: a comparative trial of two modalities for palatal closure. Plast Reconstr Surg 2001;107:9–14 10.1097/00006534-200101000-00002 11176594

[14] Kuehn DP Moller KT. Speech and language issues in the cleft palate population: the state of the art. Cleft Palate Craniofac J 2000;37:1–35

[15] John A Sell D Sweeney T. The cleft audit protocol for speech-augmented: a validated and reliable measure for auditing cleft speech. Cleft Palate Craniofac J 2006;43:272–288 10.1597/04-141.1 16681400

[16] Losee JE Hopper R Neligan P. Plastic surgery Volume 3, Craniofacial, head and neck surgery and pediatric plastic surgery, 5th ed. Elsevier; 2024. https://www.clinicalkey.com/dura/browse/bookChapter/3-s2.0-C20200011035

[17] Swanson JW Mitchell BT Cohen M. The effect of furlow palatoplasty timing on speech outcomes in submucous cleft palate. Ann Plast Surg 2017;79:156–161 10.1097/SAP.0000000000001056 28328639

[18] Ha KM Cleland H Greensmith A. Submucous cleft palate: an often-missed diagnosis. J Craniofac Surg 2013;24:878–885 10.1097/SCS.0b013e31827fef4b 23714901

[19] Pasick CM Shay PL Stransky CA. Long term speech outcomes following late cleft palate repair using the modified Furlow technique. Int J Pediatr Otorhinolaryngol 2014;78:2275–2280 10.1016/j.ijporl.2014.10.033 25468463

[20] Mathes SJ Hentz VR. Plastic surgery, 2nd ed. Vol. IV Pediatric plastic surgery. Saunders Elsevier; 2006

[21] Smith DM Vecchione L Jiang S. The Pittsburgh Fistula Classification System: a standardized scheme for the description of palatal fistulas. Cleft Palate Craniofac J 2007;44:590–594 10.1597/06-204.1 18177198

[22] Meijer M. Testing speech of children born with a cleft: a report from the speech therapy workgroup from the Dutch Association for Cleft Palate and Craniofacial Anomalies. Amsterdam: Vrije Universiteit Medisch Centrum; 2003

[23] Wijnants N Ritzen TFP Verbeek MJM, . Evaluation of speech outcomes in children with cleft palate following primary single-stage palatoplasty. *J Craniofac Surg*. 2026 10.1097/SCS.0000000000012802 PMC13764263 42059460

[24] Álvarez Carvajal DC Inostroza-Allende F Geldres Meneses MB. Speech outcomes and velopharyngeal function in children undergoing submucous cleft palate repair. J Craniofac Surg 2023;34:1766–1771 10.1097/SCS.0000000000009570 37526200

[25] Baek RM Kim BK Jeong JH. The effect of age at surgery and compensatory articulation on speech outcome in submucous cleft palate patients treated with double-opposing Z-plasty: A 10-year experience. J Plast Reconstr Aesthet Surg 2017;70:646–652 10.1016/j.bjps.2016.12.025 28325564

[26] Raby-Smith W Hall P Southby L. A retrospective analysis of speech and hearing in patients receiving surgery for submucous cleft palate. J Plast Reconstr Aesthet Surg 2023;77:123–130 10.1016/j.bjps.2022.11.003 36566640

[27] Jeon S Park JS Han M. Comparison of speech outcomes between speech therapy only and double-opposing Z-plasty combined with speech therapy in patients with submucous cleft palate. J Craniofac Surg 2024;36:1030–1034 10.1097/SCS.0000000000010385 38830053

[28] Seagle MB Patti CS Williams WN. Submucous cleft palate: a 10-year series. Ann Plast Surg 1999;42:142–148 10.1097/00000637-199902000-00006 10029477

[29] Bezuhly M Fischbach S Klaiman P. Impact of 22q deletion syndrome on speech outcomes following primary surgery for submucous cleft palate. Plast Reconstr Surg 2012;129:502e–510e 10.1097/PRS.0b013e3182402e52 22373999

[30] Pitkänen V Szwedyc A Alaluusua S. Outcomes of primary furlow double-opposing Z-plasty for the treatment of symptomatic submucous cleft palate. J Craniofac Surg 2023;34:2066–2070 10.1097/SCS.0000000000009385 PMC10521778 37221637

[31] Ritzen TFP Wijnants N Verbeek MJM. Speech therapy only versus speech therapy combined with Furlow palatoplasty for the treatment of velopharyngeal insufficiency in submucous cleft palate: a systematic review and meta-analysis. J Plast Reconstr Aesthet Surg 2025;108:93–107 10.1016/j.bjps.2025.07.014 40729976

[32] Kwinter A Dworschak-Stokan A Paradis J. Association between symptomatic submucous cleft palate and otologic disease: a retrospective review. Int J Pediatr Otorhinolaryngol 2018;115:77–81 10.1016/j.ijporl.2018.09.022 30368399

[33] Chen Y-W Philip Chen K-T Chang P-H. Is otitis media with effusion almost always accompanying cleft palate in children?: the experience of 319 Asian patients. Laryngoscope 2012;122:220–224 10.1002/lary.22425 22183637

[34] Wijnants N Widdershoven JCC van Boxel SCJ. Impact of tympanostomy tube placement on hearing outcomes in children with cleft palate-related otitis media: retrospective analysis of a primary single-stage cleft palate repair cohort. Ear Nose Throat J 2025:1455613251376792. doi:10.1177/01455613251376792 40995937

[35] Bruneel L Bettens K De Bodt M. Stages in the development and validation of a Belgian Dutch outcome tool for the perceptual evaluation of speech in patients with cleft palate. Cleft Palate Craniofac J 2020;57:43–54 10.1177/1055665619862726 31311307

[36] Chee-Williams JL Cordero KN Lien KM. Can hypernasality be reliably rated using the American English phrase sample as a part of the cleft audit protocol for speech-augmented-Americleft Modification (CAPS-A-AM) protocol? Cleft Palate Craniofac J 2025;63:10556656251339884 10.1177/10556656251339884 PMC12353658 40336290

