# Supplementary material for: Velopharyngeal Insufficiency in Submucous Cleft Palate: Importance of Combined Surgical Cleft Palate Repair and Dedicated Speech Therapy
Source: J Craniofac Surg. 2026 Jun 17;37(11-12):3129–34. doi: 10.1097/SCS.0000000000013070 (PMC13764256; doi:10.1097/SCS.0000000000013070)
Supplement: Supplementary file 1 [file scs-37-03129-s001.docx]

# Supplemental table 1: Study characteristics

| **Characteristics** | **All**  N* = 33 (%) |
| --- | --- |
| Male | 15 (45) |
| Female | 18 (55) |
| Age at diagnosis and first treatment |  |
| Median (years) | 10.7 (IQR 7.1–17.4) |
| Age at surgery |  |
| Median (years) | 4.0 years (IQR 2.6–6.1) |
| Age <18 | 29 (88) |
| Age >18 | 4 (12) |
| Prematurity |  |
| Yes | 3 (9) |
| Not reported | 14 (42) |
| Average birthweight (grams) | 3007 |
| Comorbidity^#^ | 9 (27) |
| Syndromes^#^ | 7 (21) |
| DNA-abnormality^#^ | 12 (36) |
| Consanguinity | 0 |
| Submucous cleft palate characteristics |  |
| No signs reported | 12 (36) |
| Uvula bifida | 13 (39) |
| Zona pellucida | 0 |
| Notch hard palate | 0 |
| Uvula bifida + zona pellucida | 3 (9) |
| Uvula bifida + notch hard palate | 5 (15) |
| Uvula bifida + zona pellucida + notch hard palate | 0 |
| Treatment |  |
| No treatment | 2 (6%) |
| Speech therapy only | 5 (15) |
| Surgery without postoperative speech therapy† | 4 (12) |
| Surgery with postoperative speech therapy‡ | 22 (67) |
| Ear problems |  |
| OME/AOM^#^ | 29 (88) |
| History of ventilation tubes placed | 27 (82) |
| Postoperative complications |  |
| Fistula | 3 (9) |
| Bifid uvula | 5 (15) |

*N = number of patients

^#^ OME/AOM = otitis media with effusion/acute otitis media

†Surgery with preoperative, but without postoperative speech therapy

‡Surgery with preoperative and postoperative speech therapy

\# Further specified in Supplemental table 2

# Supplemental table 2: Syndromes and anomalies

| **Characteristics** | **All**  **N*= 33 (%)** |
| --- | --- |
| **Syndromes** | 7 (21) |
| Velocardiofacial (VCF) | 3 |
| DOOR/Coffin Siris | 1 |
| Beckwith-Wiedemann | 1 |
| Horan-Beighton | 1 |
| 2q22.3-2q23.3 microdeletion | 1 |
| **Non-syndromal DNA-abnormality** | 5 (15) |
| Pierre Robin (PR) | 1 |
| ASXL2 mutation | 1 |
| SEPT9 heterozygote variation + CPLANE variant | 1 |
| DMPK mutation | 1 |
| IGFR-1 mutation + GNB2 mutation | 1 |
| **Comorbidities** | 9 (27) |
| Central nervous system | 7 |
| Plagiocephaly | 1 |
| Hydrocephaly + osteopathy striata with cranial sclerosis | 1 |
| Autism | 0 |
| Psychomotor retardation | 1 |
| Mental retardation (isolated) | 3 |
| Spastic paresis | 1 |
| Epilepsy | 1 |
| Urogenital tract | 3 |
| Abnormal kidney position | 1 |
| Hydronephrosis and ectopic dysplastic kidney | 1 |
| Solitary kidney | 1 |
| Circulatory tract | 1 |
| Atrial and ventricular septal defect | 1 |
| Lower extremity | 2 |
| Pes equinus and pes equinovarus | 1 |
| Hip dysplasia | 1 |

***** Number of patients

# Supplemental table 3: Treatments and complications

| **Surgical technique** | **Primary treatment** | **Secondary treatment** | **Simultaneous with other technique** | **With post-operative speech therapy** | **Speech therapy completed** | **Revision needed** | **Revision technique** | **Fistula^#^** | **Bifid uvula** |
| --- | --- | --- | --- | --- | --- | --- | --- | --- | --- |
| *Intra-velar veloplasty* | 3 | 0 | 0 | 2 | 1 | 0 | N/A | Type 2 | 0 |
| *Cranially based pharyngoplasty* | 3 | 3 | 1 | 2 | 3 | 0 | N/A | 0 | 0 |
| *Caudally based pharyngoplasty* | 1 | 0 | 0 | 1 | 1 | 1 | Furlow with cranially based pharyngoplasty | Type 2 | 0 |
| *Furlow* | 18 | 1 | 1 | 16 | 13 | 1 | Cranially based pharyngoplasty | Type 3 | 5 |
| *Furlow with buccal fat pad flap* | 1 | 0 | 0 | 0 | 0 | 0 | N/A | 0 | 0 |
| *AFT* posterior pharynx wall + upper side soft palate* | 1 | 0 | 0 | 1 | 1 | 1 | Cranially based pharyngoplasty | 0 | 0 |
| **Total** | 27 | 4 | 2 | 22 | 19 | 3 |  | 3 | 5 |

^#^Fistula type according to the Pittsburgh Fistula Classification system

*AFT = autologous fat transplantation

‡Total = the total primary surgical techniques do not match the number of patients receiving surgery, as the simultaneously performed surgical treatments are registered separately in the table.

# Supplemental Table 4: Cleft Type Characteristics (CTCs) at first and last speech evaluation

|  | | **Speech therapy only group** | | **Surgery combined with speech therapy group** | |
| --- | --- | --- | --- | --- | --- |
|  |  | **First evaluation** | **Last evaluation** | **First evaluation** | **Last evaluation** |
| **Anterior Oral CTCs** | **Dentalisation/ inter-dentalisation** |  |  |  |  |
|  | Absent | 1 | 11 | 11 | 25 |
|  | ≤ Two consonants affected | 1 | 2 | 2 | 0 |
|  | ≥ Three consonants affected | 2 | 3 | 3 | 0 |
|  | **Lateralisation** |  |  |  |  |
|  | Absent | 4 | 16 | 16 | 25 |
|  | ≤ Two consonants affected | 0 | 0 | 0 | 0 |
|  | ≥ Three consonants affected | 0 | 0 | 0 | 0 |
|  | **Palatalisation** |  |  |  |  |
|  | Absent | 4 | 16 | 16 | 25 |
|  | ≤ Two consonants affected | 0 | 0 | 0 | 0 |
|  | ≥ Three consonants affected | 0 | 0 | 0 | 0 |
| **Posterior Oral CTCs** | **Double articulation** |  |  |  |  |
|  | Absent | 4 | 16 | 16 | 25 |
|  | ≤ Two consonants affected | 0 | 0 | 0 | 0 |
|  | ≥ Three consonants affected | 0 | 0 | 0 | 0 |
|  | **Backed to velar/uvular** |  |  |  |  |
|  | Absent | 4 | 16 | 16 | 25 |
|  | ≤ Two consonants affected | 0 | 0 | 0 | 0 |
|  | ≥ Three consonants affected | 0 | 0 | 0 | 0 |
| **Non-Oral CTCs** | **Pharyngeal articulation** |  |  |  |  |
|  | Absent | 4 | 15 | 15 | 25 |
|  | ≤ Two consonants affected | 0 | 1 | 1 | 0 |
|  | ≥ Three consonants affected | 0 | 0 | 0 | 0 |
|  | **Glottal articulation** |  |  |  |  |
|  | Absent | 4 | 14 | 14 | 24 |
|  | ≤ Two consonants affected | 0 | 1 | 1 | 1 |
|  | ≥ Three consonants affected | 0 | 1 | 1 | 0 |
|  | **Active nasal fricatives** |  |  |  |  |
|  | Absent | 4 | 14 | 14 | 24 |
|  | ≤ Two consonants affected | 0 | 2 | 2 | 1 |
|  | ≥ Three consonants affected | 0 | 0 | 0 | 0 |
|  | **Glottal amplification** |  |  |  |  |
|  | Absent | 4 | 16 | 16 | 25 |
|  | ≤ Two consonants affected | 0 | 0 | 0 | 0 |
|  | ≥ Three consonants affected | 0 | 0 | 0 | 0 |
| **Passive CTCs** | **Weak and/or nasalised consonants** |  |  |  |  |
|  | Absent | 4 | 16 | 16 | 25 |
|  | ≤ Two consonants affected | 0 | 0 | 0 | 0 |
|  | ≥ Three consonants affected | 0 | 0 | 0 | 0 |
|  | **Nasal realisation of plosives and/or suspected passive nasal fricatives** |  |  |  |  |
|  | Absent | 2 | 16 | 16 | 23 |
|  | ≤ Two consonants affected | 1 | 0 | 0 | 2 |
|  | ≥ Three consonants affected | 1 | 0 | 0 | 0 |
|  | **Gliding of fricatives/affricates** |  |  |  |  |
|  | Absent | 4 | 16 | 16 | 25 |
|  | ≤ Two consonants affected | 0 | 0 | 0 | 0 |
|  | ≥ Three consonants affected | 0 | 0 | 0 | 0 |

# Supplemental Table 5: Change in CAPS-A Dutch speech parameters in pre- and post-surgical speech evaluation

|  | **Surgery combined with speech therapy group** | | | | | **Speech therapy only group** | |
| --- | --- | --- | --- | --- | --- | --- | --- |
|  | **Preoperative** | | | | **Postoperative** | **First evaluation** | **Last evaluation** |
| **Speech intelligibility** | ^#^p=0.005;0.012;1.000 | | | | | *p=1.000 | |
| 0 | 0 | | | | 4 | 0 | 0 |
| 1 | 2 | | | | 11 | 2 | 2 |
| 2 | 9 | | | | 8 | 2 | 1 |
| 3 | 5 | | | | 2 | 0 | 1 |
| 4 | 0 | | | | 0 | 0 | 0 |
| Not evaluated | 10 | | | | 1 | 3 | 3 |
| **Hypernasality** | p=0.005;0.007;1.000 | | | | | p=0.346 | |
| 0 | 1 | | | | 13 | 0 | 0 |
| 1 | 0 | | | | 1 | 0 | 1 |
| 2 | 3 | | | | 6 | 2 | 1 |
| 3 | 6 | | | | 3 | 1 | 2 |
| 4 | 6 | | | | 2 | 1 | 0 |
| Not evaluated | 10 | | | | 1 | 3 | 3 |
| **Hyponasality** | | | | p=1.000;1.000;1.000 | | p=1.000 | |
| 0 | 15 | | | | 23 | 4 | 4 |
| 1 | 1 | | | | 2 | 0 | 0 |
| 2 | 0 | | | | 0 | 0 | 0 |
| Not evaluated | 10 | | | | 1 | 3 | 3 |
| **Nasal emission** | | | p=0.374;0.345;1.000 | | | p=1.000 | |
| 0 | 9 | | | | 16 | 1 | 1 |
| 1 | 1 | | | | 5 | 1 | 1 |
| 2 | 6 | | | | 4 | 2 | 2 |
| Not evaluated | 10 | | | | 1 | 3 | 3 |
| **Nasal turbulence** | | p=0.530;0.299;1.000 | | | | p=1.000 | |
| 0 | 6 | | | | 12 | 3 | 3 |
| 1 | 5 | | | | 11 | 0 | 0 |
| 2 | 5 | | | | 2 | 1 | 1 |
| Not evaluated | 10 | | | | 1 | 3 | 3 |

^#^ P-value: represents the significance in change of speech parameters before and after treatment by surgery combined with speech therapy for the overall cohort; non-syndromic patients only; and syndromic patients only respectively

* P-value: represents the significance in change of speech parameters before and after treatment by speech therapy only

# Supplemental Table 6: Nasometry and mirror test results from speech assessments

|  | **Surgery combined with speech therapy group** | | **Speech therapy only group** | |
| --- | --- | --- | --- | --- |
|  | **Preoperative** | **Postoperative** | **First evaluation** | **Last evaluation** |
| **Nasometry** | | | | |
| Normal | 1 | 3 | 1 | 1 |
| Abnormal | 5 | 7 | 2 | 1 |
| Not evaluated | 20 | 16 | 1 | 2 |
| **Mirror test** | | | | |
| Absent | 0 | 2 | 1 | 1 |
| Present | 4 | 7 | 1 | 1 |
| Not evaluated | 22 | 19 | 2 | 2 |
